# Predictors of Clinical Outcome in Women with Pelvic Organ Prolapse Who Underwent Transvaginal Mesh Reconstruction Surgery

**DOI:** 10.3390/medicina58020148

**Published:** 2022-01-19

**Authors:** Ting-Hsuan Lin, Fung-Chao Tu, Ho-Hsiung Lin, Sheng-Mou Hsiao

**Affiliations:** 1 Department of Obstetrics and Gynecology, Far Eastern Memorial Hospital, Banqiao, New Taipei 220409, Taiwan; lindonut713@gmail.com (T.-H.L.); fctu77@yahoo.com.tw (F.-C.T.); hhlin@ntuh.gov.tw (H.-H.L.); 2 Department of Obstetrics and Gynecology, National Taiwan University College of Medicine and the Hospital, Taipei 100225, Taiwan; 3 Graduate School of Biotechnology and Bioengineering, Yuan Ze University, Taoyuan 320315, Taiwan

**Keywords:** pelvic organ prolapse, urinary incontinence, stress, urinary bladder, overactive, surgical mesh

## Abstract

*Background and Objectives*: To identify the predictors of clinical outcomes in women with pelvic organ prolapse (POP) who underwent transvaginal reconstruction surgery, especially with transobturator mesh fixation or sacrospinous mesh fixation. *Materials and Methods*: All women with POP who underwent transvaginal reconstruction surgery, especially with transobturator mesh fixation or sacrospinous mesh fixation, were reviewed. *Results:* Between January 2011 and May 2019, a total of 206 consecutive women were reviewed, including 68 women receiving POP reconstruction with transobturator mesh fixation and 138 women who underwent POP reconstruction with sacrospinous mesh fixation. The least experienced surgeon (hazard ratio = 804.6) and advanced stage of cystocele (hazard ratio = 8.80) were the predictors of POP recurrence, especially those women with stage 4 of cystocele. Young age (hazard ratio = 0.94) was a predictor for mesh extrusion, especially those women with age ≤67 years. Follow-up interval (odds ratio = 1.03, *p* = 0.02) was also an independent predictor of mesh extrusion. High maximum flow rate (Qmax, hazard ratio = 1.03) was the sole predictor of postoperative stress urinary incontinence, especially those women with Qmax ≥19.2 mL/s. Preoperative overactive bladder syndrome (hazard ratio = 3.22) were a predictor for postoperative overactive bladder syndrome. In addition, overactive bladder syndrome rate improved after surgery in the sacrospinous group (*p* = 0.0001). Voiding dysfunction rates improved after surgery in both sacrospinous and transobturator groups. *Conclusions:* Predictors of clinical outcome in women who underwent transvaginal POP mesh reconstruction are identified. The findings can serve as a guide for preoperative consultation of similar procedures.

## 1. Introduction

Pelvic organ prolapse (POP) includes prolapse of the anterior, apical, and posterior compartments. Anterior vaginal wall is the vaginal site most commonly affected by prolapse. Instead of an isolated defect, anterior vaginal wall prolapse is highly associated with apical prolapse [1,2]. Concomitant apical suspension during anterior vaginal wall repair was demonstrated to reduce the recurrence rate of pelvic organ prolapse (POP) [3].

Higher burden of climacteric symptoms is associated with POP compared without POP [4]. Thus, the clinical outcome of POP reconstruction is an important issue in postmenopausal women. POP reconstruction can be performed by transvaginal and transabdominal approaches. Transvaginal mesh reconstruction for anterior/apical POP includes transobturator and sacrospinous mesh fixations, such as the Perigee (American Medical Systems, Minnetonka, MN, USA) and Uphold (Boston Scientific, Natick, MA, USA) systems [5,6,7]. Despite the above systems are not available in the market owing to the FDA warning [8]. However, some self-tailored vaginal mesh [9,10,11,12] and transvaginal mesh kits, such as Calistar system (Promedon, Argentine [13,14], Surelift system (Neomedic International, Terrassa, Spain [15]), Pelvimesh system (Herniamehs, Italy [16]), and Seratom^®^ PA system (Seratex^®^ PA B2 type, Serag-Wiessner KG, Naila, Germany [17]) are still in use. In the era of native tissue repair, autologous rectus fascia or fascia lata are used for transobturator or sacrospinous fixation [18,19,20,21]. Thus, the data of predictors of clinical outcome after transvaginal mesh reconstruction should be important for preoperative consultation. 

To our knowledge, there was only one study mentioned about the comparison of POP surgery between transobturator and sacrospinous mesh fixation, and the predictor of clinical outcome was not analyzed in the study [22]. Therefore, the primary objective of this study was to identify predictors for clinical outcome in women who underwent POP reconstruction, especially with transobturator or sacrospinous mesh fixation. 

## 2. Methods

Medical records of all consecutive women with Pelvic Organ Prolapse Quantification stage II or higher anterior/apical compartment prolapse, who were admitted to the department of Obstetrics and Gynecology of a tertiary referral center for POP reconstruction were reviewed. Those patients who did not undergo transobturator (i.e., the Perigee system) or sacrospinous (i.e., the Uphold system) mesh fixation were excluded in this study. In general, uncontrolled diabetes is a contraindication of vaginal mesh surgery in our hospital. The research ethics review committee of this hospital approved this study. 

Transobturator mesh fixation was available between January 2011 and October 2016; however, sacrospinous mesh fixation was available between June 2015 and May 2019 in the hospital. That is, patients received transobturator mesh fixation between January 2011 and June 2015, and patients received sacrospinous mesh fixation between October 2016 and May 2019. Between June 2015 and October 2016, the choice of sacrospinous or transobturator mesh fixation was made at each surgeon’s discretion. 

### 2.1. Operative Technique

#### 2.1.1. Transobturator Mesh Fixation

After hydrodissection, a vertical midline incision was made on the anterior vaginal wall. The vaginal epithelium and the full-thickness muscularis layer were dissected from the bladder wall. The vesicovaginal space was opened bilaterally until the plane near the ischial spine. Frequently, the anterior wall prolapse was plicated with absorbable sutures to reduce the area of the cystocele. Four cutaneous incisions were made; 2 superior incisions were made at the level of the clitoris at the upper medial edge of the obturator foramen and 2 inferior incisions were made 3 cm inferior and 2 cm lateral to the superior incisions. Superior trocars were inserted through the incision wound, penetrating the subcutaneous tissue, passing through the obturator membrane, and emerging from the vaginal incision wound with finger guidance. With the similar method, the inferior mesh arms were attached to the pelvic side wall at the level of the arcus tendineus fasciae pelvis near the ischial spine. The central part of the mesh was placed under the bladder, laid flat on the anterior vagina wall, and fixed loosely. The vaginal incision wound was closed with two layers of delayed absorbable suture [7]. 

#### 2.1.2. Sacrospinous Mesh Fixation

After hydrodissection, a longitudinal midline vaginal incision was made with blunt dissection of the vaginal mucosa from its underlying fascia until identifying the sacrospinous ligament. With the aid of the Capio suture capturing device (Boston Scientific, Natick, MA, USA), the mesh arms were introduced and fixed at the bilateral sacrospinous ligaments. Instead of direct visualization, the location of mesh fixation was identified by palpation (about two finger breaths medial to the ischial spine). The central part of the mesh was sutured to the bladder wall and paracervical ring or vaginal vault. After adjusting the tension of the mesh, the incision wound was closed with two layers of delayed absorbable sutures [23].

Medical records, including obstetric and gynecologic history, body mass index, systemic disease, previous urogynecologic surgery history, coexistent overactive bladder syndrome (OAB), 20 min pad test, and urodynamic studies, were reviewed. In general, patients were requested to visit the outpatient clinic 7 days, 14 days, 1 month, and 3 months after surgery, and then 6-monthly thereafter. Stress urinary incontinence (SUI) was defined as the complaint of involuntary loss of urine on effort, physical exertion, sneezing, or coughing [24]. Postoperative OAB was defined if the patient received antimuscarinics or beta-3 agonist for OAB treatment during follow-up. The date of first dose of antimuscarinics or beta-3 agonist was defined as the onset date of postoperative OAB. The date of SUI-free or OAB-free interval was calculated from the date of surgery to the date of documented SUI, OAB or the date of last follow-up. Detrusor overactivity was defined if evidence of involuntary detrusor contractions during filling cystometry [24]. Voiding dysfunction (VD) was defined as the following symptoms during or following the act of micturition, including hesitancy, slow steam, intermittency, straining to void, spraying of urinary stream, feeling of incomplete emptying, need to immediately revoid, postmicturition leakage, position-dependent micturition, dysuria or urinary retention [24]. 

Multichannel urodynamic equipment (Life-Tech, Houston, TX, USA) with computer analysis and Urovision (Urolab Janus System V, Houston) was used for women with coexistent lower urinary tract symptoms or excluding occult urodynamic stress incontinence. All terminology conformed to the standards recommended by the International Continence Society and Urodynamic Society [24]. All data were interpreted by a single observer to avoid interobserver variability.

The Stata software program (Version 11.0; Stata Corp, College Station, TX, USA) was used for statistical analyses. Chi-square test, Fisher’s exact test, Wilcoxon rank-sum test, or McNemar’s test were employed for statistical analysis. The survival curve was estimated using the Kaplan–Meier method. Multivariable Cox proportional hazards model was performed by using all variables with *p* < 0.10 in the univariate analysis [25]. A *p* value of less than 0.05 was considered statistically significant. The receiver operating characteristic curve (ROC) analysis was performed to identify the optimal cut-off value for differentiation. 

## 3. Results

Between January 2011 and May 2019, a total of 206 consecutive women were reviewed, including 68 women who received POP reconstruction with transobturator mesh fixation and 138 women who underwent POP reconstruction with sacrospinous mesh fixation. The baseline characteristics of the patients are listed in Table 1. The median postoperative follow-up interval of the transobturator group (6.8 months, 25–75 interquartile range = 2.4 to 30.8 months) was longer than the sacrospinous group (4.7 months, 25–75 interquartile range = 2.5 to 11.6 months, *p* = 0.01). A total of 161 (78.2%) patients underwent preoperative urodynamic studies. Most patients were menopausal. Except ≥stage II uterine prolapse and rectocele rates, detrusor pressure at maximum flow rate, vaginal total hysterectomy, posterior colporrhaphy and follow-up interval, there were no between-group differences in the other baseline characteristics (Table 1). There was no significant between-group difference in operative time and intraoperative blood loss (Table 1).

Five surgeons were involved in this study (Table 1). Between June 2015 and October 2016, fifty-seven patients underwent sacrospinous or transobturator mesh fixation according to each surgeon’s discretion. However, two surgeons preferred to perform transobturator mesh fixation (transobturator/sacrospinous case ratio = 11/4), and three surgeons tended to perform sacrospinous mesh fixation (transobturator/sacrospinous case ratio = 8/34) (*p* < 0.001). In addition, the presence of apical prolapse was a negative predictor of the use of transobturator mesh fixation (odds ratio = 0.26, 95% confidence interval (CI) = 0.09 to 0.76, *p* = 0.01). That is, those patients without concomitant apical prolapse tended to undergo transobturator mesh fixation.

**Figure 1:**
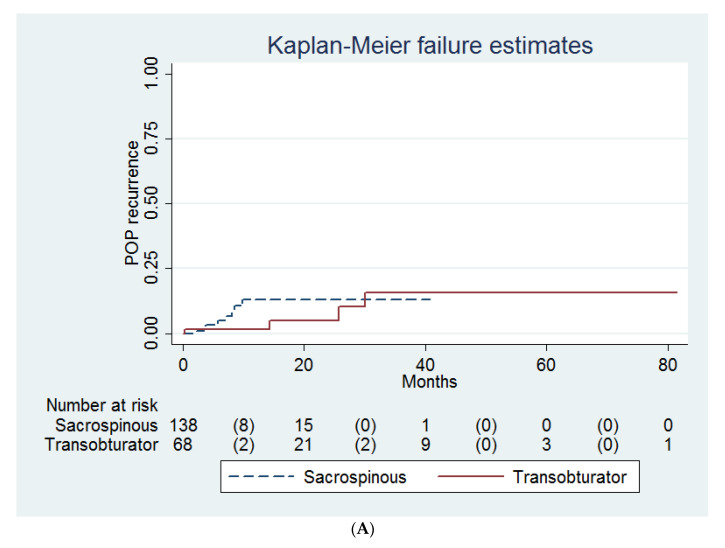

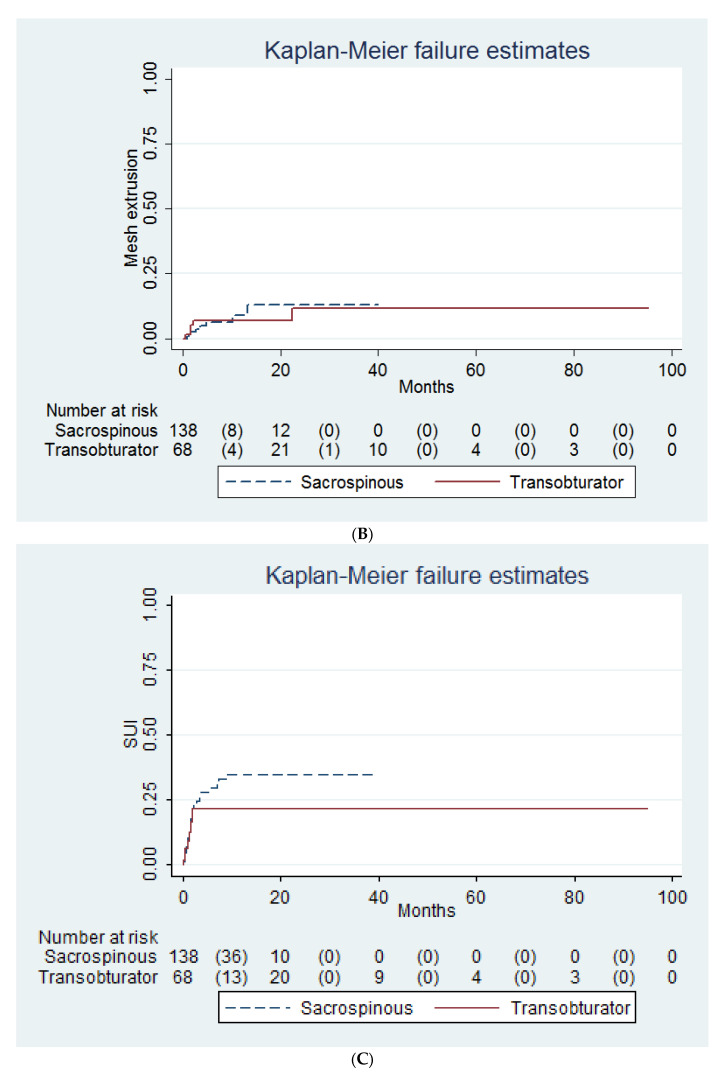

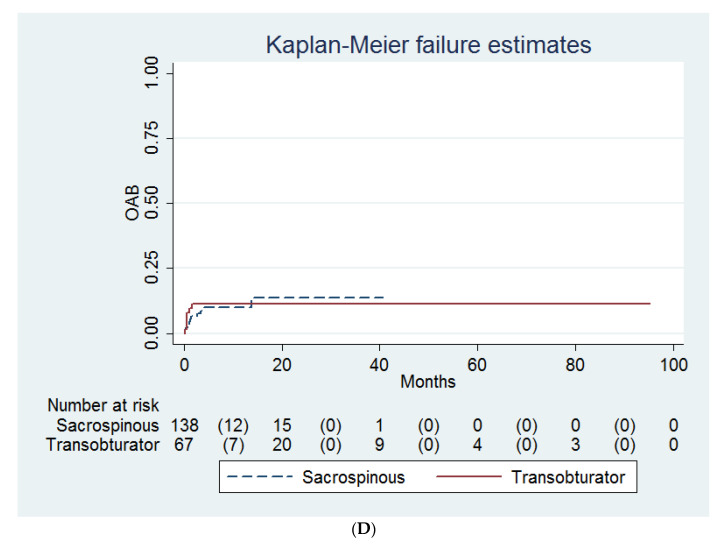
Probabilities of (**A**) recurrence of pelvic organ prolapse (POP), (**B**) mesh extrusion, (**C**) stress urinary incontinence (SUI) and (**D**) overactive bladder syndrome (OAB) after the transobturator (*n* = 68) or sacrospinous (*n* = 138) mesh fixations.

Probabilities of POP recurrence (Figure 1A), mesh extrusion (Figure 1B), postoperative SUI (Figure 1C), and postoperative OAB (Figure 1D) did not differ between the sacrospinous and transobturator groups (log-rank test, *p* = 0.42, 0.97, 0.24 and 0.75, respectively). 

**Figure 2:**
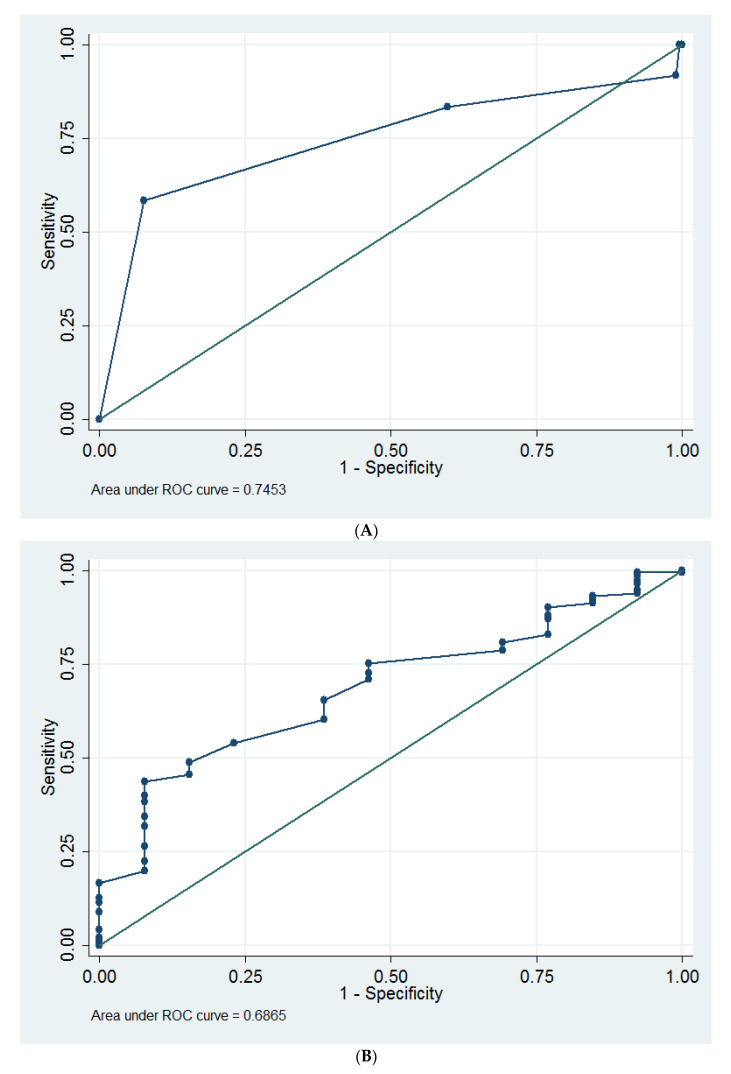
The receiver operating characteristic curves for (**A**) stage of cystocele as a predictor of recurrence of pelvic organ prolapse, and (**B**) age as a predictor of mesh extrusion.

Multivariable Cox proportional hazards model revealed that the stage of cystocele (hazard ratio = 4.56) and surgeon E (hazard ratio = 804.6) were the independent predictors of POP recurrence (Table 2). Stage of cystocele ≥ 4 was determined as an optimum cut-off value for predicting POP recurrence, which had an area under the ROC curve of 0.74 (95% CI = 0.56 to 0.93; sensitivity = 58.3%, specificity = 92.5%, Figure 2A).

Multivariable Cox proportional hazards model revealed that age (hazard ratio = 0.94) was the only predictor of mesh extrusion (Table 2). Age ≤ 67 years was determined as an optimum cut-off value for predicting a higher rate of mesh extrusion, which had an area under the ROC curve of 0.69 (95% CI = 0.56 to 0.82; sensitivity = 43.5%, specificity = 92.3%, Figure 2B). If we used multivariable logistic regression analysis, the follow-up interval (odds ratio = 1.03, 95% CI = 1.02 to 1.05, *p* = 0.02) was also an independent predictor of mesh extrusion in additional to young age (odds ratio = 0.94, 95% CI = 0.88 to 1.00, *p* = 0.04). 

**Figure 3:**
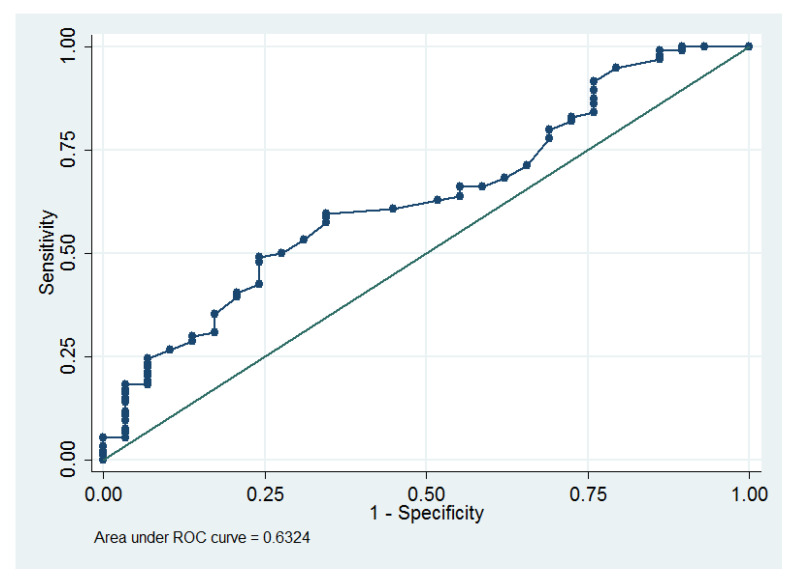
The receiver operating characteristic curve for the maximum flow rate as a predictor of postoperative stress urinary incontinence.

Multivariable Cox proportional hazards model also revealed that Qmax (hazard ratio = 1.03) was the sole predictor of postoperative SUI (Table 3). Qmax ≥ 19.2 mL/s was determined as an optimum cut-off value for predicting postoperative SUI, which had an area under the ROC curve of 0.59 (95% CI = 0.48 to 0.69; sensitivity = 62.5%, specificity = 55.1%, Figure 3).

Multivariable Cox proportional hazards model revealed that preoperative OAB (hazard ratio = 3.22) was the sole predictor for postoperative OAB (Table 3). 

Univariate logistic regression analysis did not reveal any predictors for postoperative VD (Table 4).

Comparison of baseline and postoperative lower urinary tract symptoms was shown in Table 5. In both groups, SUI rates did not change after surgery in women without concomitant mid-urethral sling procedures. However, OAB improved after surgery in the sacrospinous group (*p* = 0.0001). In addition, VD improved after surgery in both groups (Table 5).

## 4. Discussion

In this study, the method of POP reconstruction (i.e., sacrospinous versus transobturator mesh fixation) is not a predictor for POP recurrence, mesh extrusion, and postoperative SUI, OAB and VD (Figure 1A–D, Table 2, Table 3 and Table 4). Similarly, Lo et al. found that sacrospinous fixation (i.e., the Elevate system, American Medical Systems, Minnetonka, MN, USA) had a similar objective and subjective cure rate, compared with transobturator fixation (i.e., the Perigee system) [22]. Kato et al. also reported that the reoperation rates due to prolapse recurrence and mesh exposure rates did not differ between the Prolift-type (i.e., anterior mesh with transobturator arms, posterior mesh with sacrospinous arms) and Uphold-type (i.e., anterior mesh with sacrospinous arms) groups [26]. 

Cystocele stage was a predictor for POP recurrence (hazard ratio = 4.56, Table 3). Similarly, Vergeldt et al. reported that cystocele stage could predict cystocele recurrence [27]. Recently, a meta-analysis also revealed preoperative stage 2–4 (odds ratio = 2.11, *p* < 0.001) was one significant predictor of POP recurrence [28]. We also found that stage 4 of cystocele was an optimal cut-off point for predicting POP recurrence (Figure 2A), and this finding could be used as a reference for preoperative consultation.

In our study, the least experienced surgeon (i.e., only three cases were performed by surgeon E) was a risk factor for POP recurrence (hazard ratio = 804.60, *p* < 0.001, Table 2). Similarly, Long et al. also reported that the POP failure rate was higher during the first 50 cases, compared with the subsequent cases (5% vs. 1.6%, *p* = 0.043) [29]. Price et al. reported that the majority of POP failures occur within the first three years following the initial operation [30]. Nonetheless, Nüssler et al. reported that the high POP recurrence rate was not due to insufficient experience of the surgeons performing the operation [31].

Young age was a predictor for mesh extrusion (hazard ratio = 0.94, *p* = 0.04, Table 2); and age ≤ 67 years was determined as an optimum cut-off value for predicting mesh extrusion (Figure 2B). Similarly, Khrucharoen et al. reported that menopause was not a predictor for mesh extrusion (odds ratio = 1.385, *p* = 0.15) [32]. The reason for young age as a predictor for mesh extrusion was unknown. However, age-related complaints (e.g., decreased lubrication and orgasmic difficulties) could result in sexual dysfunction [33,34]. Asian middle-aged and older women tend to report less frequent sexual activity [35]. Thus, sexual dysfunction in aged women might be responsible for a lower rate of mesh extrusion. It is worth mentioning that age was not significantly associated with mesh extrusion in Ehsani et al.’s study [36]. In addition, it is worth mentioning that the follow-up time interval (odds ratio = 1.03, *p* = 0.02) was also an independent predictor of mesh extrusion. The mesh extrusion rate might increase with time.

Qmax was a predictor for postoperative SUI (hazard ratio = 1.03, *p* = 0.04, Table 4), especially for those women with Qmax ≥ 19.2 mL/s (Figure 3). Similarly, Kawaguchi et al. reported that high flow rate was associated with postoperative SUI [37]. High flow rate was associated with SUI [38,39,40]. Therefore, it seems reasonable that Qmax is a predictor of postoperative SUI.

Preoperative OAB (hazard ratio = 3.22, *p* = 0.01) was an independent predictor for postoperative OAB (Table 4), which is in line with the existing studies. de Boer TA et al. also found that the absence of preoperative OAB was the best predictor for the absence of postoperative OAB [41,42]. 

OAB improved after sacrospinous mesh fixation (*p* = 0.0001, Table 5). Similarly, several studies also found that an improvement of OAB after POP construction [43,44,45], and a reduction of 50% of urinary urgency incontinence could be expected [45]. VD improved after transobturator or sacrospinous mesh fixation (Table 5). POP surgery can improve anatomic support of the anterior compartment, thus improving VD.

Limitations of this study included a retrospective nature and limited sample size. In addition, different between-group follow-up time intervals and different surgical experience of the surgeons may bias the results. Besides, an average of two cases per month in this hospital might be not enough for surgeons to achieve surgical proficiency in POP surgery, and this can result in the bias. In general, patients with anterior compartment prolapse but without apical prolapse might be suitable to receive transobturator mesh fixation. We also found that the presence of apical prolapse was a negative predictor of the use of transobturator mesh fixation (Odds ratio = 0.26, *p* = 0.01); however, this finding might bias our results. Despite the Uphold system and the Perigee system are not available currently. However, some similar commercial kits, self-tailored meshes, and autologous fasciae remain in use for transvaginal POP reconstruction [9,10,11,12,13,14,15,16,17,18,19,20,21]. Thus, our results could provide as a guide for perioperative consultation, even in the era of native tissue repair.

## 5. Conclusions

Predictors of clinical outcome in women who underwent transvaginal POP reconstruction are identified. The above results can serve as a guide for preoperative consultation.

## Figures and Tables

**Table 1:** Baseline and peri-operative data of women who underwent surgery with transobturator or sacrospinous mesh fixation (*n* = 206).

Variables	Transobturator (*n* = 68)	Sacrospinous (*n* = 138)	† *p*
Age (years)	64.1 ± 8.0	64.4 ± 9.9	0.56
Menopause	65 (96)	120 (87)	0.08
BMI (kg/m^2^)	25.0 ± 3.0	24.8 ± 3.7	0.50
Parity	3.1 ± 1.2	3.1 ± 1.2	0.93
Diabetes	15 (22)	42 (30)	0.21
Hypertension	41 (60)	64 (46)	0.06
Prior hysterectomy	10 (15)	14 (10)	0.34
Prior POP surgery	6 (9)	10 (7)	1.00
≥stage II cystocele	67 (99)	131 (95)	0.67
≥stage II uterine prolapse	47 (69)	126 (91)	0.005
≥stage II rectocele	30 (44)	88 (65)	0.005
Pad weight (g)	17.5 ± 39.5	24.8 ± 52.9	0.48
Qmax (mL/s)	21.9 ± 10.9	19.8 ± 11.5	0.26
Voided volume (mL)	292 ± 164	261 ± 156	0.25
Post-void residual (mL)	121 ± 84	113 ± 81	0.62
PdetQmax (cmH_2_O)	27.0 ± 17.1	35.1 ± 19.2	0.01
Detrusor overactivity	14 (21)	18 (13)	0.052
MUCP (cmH_2_O)	60.1 ± 32.0	72.3 ± 44.8	0.10
SUI	28 (37)	62 (45)	0.76
OAB	13 (19)	35 (24)	0.64
VD	15 (22)	45 (33)	0.12
VTH	29 (43)	39 (28)	<0.001
MUS	7(10)	20 (14)	0.40
Posterior colporrhaphy	37 (54)	109 (79)	<0.001
Operative time (mins)	108 ± 39	119 ± 36	0.65
Blood loss (mL)	101 ± 104	142 ± 211	0.17
Perioperative Complications			
Bladder perforation	0 (0)	1 (1)	1.00
Massive bleeding	0 (0)	1 (1)	1.00
Clavien-Dindo classification			
Grade II	0 (0)	1 (1)	1.00
Grade IIIb	0 (0)	1 (1)	1.00
Follow-up interval (months)	19.1 ± 23.3	8.2 ± 8.7	0.01
Recurrence of POP	4 (6)	8 (2)	0.42 ‡
Mesh extrusion	5 (7)	8 (9)	0.97 ‡
Dysuria/UTI	7 (10)	9 (2)	0.34
De novo dyspareunia	1 (1)	1(1)	0.55
Surgeon			
A	19 (28)	86 (62)	<0.001
B	33 (49)	26 (19)	
C	7 (10)	23 (17)	
D	9 (13)	0 (0)	
E	0 (0)	3 (2)	

Values are expressed as mean ± standard deviation or number (percentage). BMI = body mass index. MUCP = maximum urethral closure pressure. MUS = mid-urethral sling. OAB = overactive bladder syndrome. PdetQmax = detrusor pressure at maximum flow rate. POP = pelvic organ prolapse. Qmax = maximum flow rate. SUI = stress urinary incontinence. UTI = urinary tract infection. VD = voiding dysfunction. VTH = vaginal total hysterectomy. † Wilcoxon rank sum test, chi-square test, or Fisher’s exact test. ‡ Log-rank test.

**Table 2:** Factors predicting recurrence of pelvic organ prolapse and mesh extrusion (*n* = 206).

	Recurrence of POP				Mesh Extrusion			
	Univariate		Multivariable		Univariate		Multivariable	
Variables	HR (95% CI)	*p* †	HR (95% CI)	*p* ‡	HR (95% CI)	*p* †	HR (95% CI)	*p* ‡
Transobturator method	0.60 (0.17, 2.11)	0.43	-	-	0.98 (0.31, 3.08)	0.97	-	-
Age (years)	1.03 (0.97, 1.09)	0.40	-	-	0.94 (0.89, 1.00)	0.04	0.94 (0.89, 1.00)	0.04
Menopause	6.94 × 10^15^ (0, infinity)	1.00	-	-	1.17 (0.15, 9.09)	0.88	-	-
Parity	1.17 (0.76, 1.80)	0.48	-	-	1.04 (0.68, 1.61)	0.84	-	-
BMI (kg/m^2^)	1.07 (0.92, 1.24)	0.39	-	-	0.98 (0.84, 1.14)	0.80	-	-
Hypertension	1.41 (0.45, 4.46)	0.56	-	-	1.64 (0.53, 5.01)	0.39	-	-
Diabetes	1.04 (0.28, 3.86)	0.95	-	-	1.31 (0.40, 4.26)	0.66	-	-
Prior hysterectomy	0.57 (0.07, 4.46)	0.59	-	-	0.61 (0.08, 4.72)	0.64	-	-
Prior POP surgery	1.21 (0.16, 9.43)	0.85	-	-	1.10 (0.14, 8.46)	0.93	-	-
Cystocele stage	6.17 (2.25, 16.91)	<0.001	8.80 (2.15, 36.09)	0.003	1.01 (0.44, 2.29)	0.99	-	-
Apical prolapse stage §	2.78 (1.44, 5.33)	0.002	-	-	0.74 (0.46, 1.19)	0.22	-	-
Pad weight (g)	1.00 (0.99, 1.02)	0.45	-	-	1.00 (0.99, 1.01)	0.90	-	-
Qmax (mL/s)	1.03 (0.98, 1.08)	0.29	-	-	0.99 (0.94, 1.04)	0.71	-	-
Voided volume (mL)	1.00 (1.00, 1.00)	0.99	-	-	1.00 (1.00, 1.00)	0.30	-	-
Post-void residual (mL)	1.01 (1.00, 1.01)	0.16	-	-	1.01 (1.00, 1.01)	0.11	-	-
PdetQmax (cmH_2_O)	0.99 (0.95, 1.05)	0.81	-	-	0.98 (0.93, 1.02)	0.26	-	-
Detrusor overactivity	0.93 (0.20, 4.36)	0.92	-	-	0.89 (0.19, 4.14)	0.89	-	-
MUCP(cmH_2_O)	1.01 (0.99, 1.02)	0.28	-	-	1.00 (0.98, 1.01)	0.74	-	-
SUI	0.87 (0.26, 2.88)	0.81	-	-	2.03 (0.68, 6.04)	0.21	-	-
OAB	1.06 (0.29, 3.94)	0.93	-	-	0.96 (0.26, 3.47)	0.95	-	-
VD	3.16 (1.02, 9.84)	0.047	2.77 (0.76, 10.10)	0.12	0.81 (0.22, 2.94)	0.75	-	-
VTH	0.24 (0.03, 1.86)	0.17	-	-	0.49 (0.11, 2.23)	0.36	-	-
Mid-urethral sling	1.31 (0.29, 6.03)	0.73	-	-	0.54 (0.07, 4.15)	0.55	-	-
Surgeon ¶								
A (reference)	1.00	-	1.00	-	1.00	-	-	-
B	9.,95 (1.19, 83.1)	0.03	3.90 (0.44, 34.43)	0.22	1.79 (0.52, 6.19)	0.36	-	-
C	14.1 (1.57, 127.08)	0.02	2.90 (0.21, 39.88)	0.43	1.90 (0.45, 7.98)	0.38	-	-
D	8.66 × 10^−19^ (-, -)	-	9.89 × 10^−19^ (-, -)	-	2.01 × 10^−15^ (0, infinity)	1.00	-	-
E	36.07 (2.20, 591.54)	0.01	804.60 (21.63, 29,924.48)	<0.001	2.00 × 10^−15^ (0, infinity)	1.00	-	-

CI = confidence interval. HR = hazard ratio. The other abbreviations are the same as in Table 1. † Cox proportional hazards model. ‡ Multivariable Cox proportional hazards modeling was performed using all variables with *p* < 0.10 in the univariate analysis. § Owing to a significant correlation between cystocele stage and apical prolapse stage (Spearman’s rho = 0.39, *p* < 0.0001), apical prolapse stage was excluded in the multivariable Cox proportional hazards modeling for predicting recurrence of pelvic organ prolapse. ¶ Experienced POP surgical cases: surgeon A > surgeon B > surgeon C > surgeon D > surgeon E.

**Table 3:** Factors predicting postoperative stress urinary incontinence and overactive bladder syndrome (*n* = 206).

	Postoperative SUI				Postoperative OAB			
	Univariate		Multivariable		Univariate		Multivariable	
Variables	HR (95% CI)	*p* †	HR (95% CI)	*p* ‡	HR (95% CI)	*p* †	HR (95% CI)	*p* ‡
Transobturator fixation	0.69 (0.36, 1.29)	0.24	-	-	1.17 (0.46, 2.98)	0.75	-	-
Age (years)	1.01 (0.98, 1.04)	0.74	-	-	1.02 (0.97, 1.07)	0.47	-	-
Menopause	0.91 (0.36, 2.29)	0.84	-	-	1.95 (0.26, 14.63)	0.52	-	-
Parity	0.92 (0.72, 1.17)	0.48	-	-	0.91 (0.63, 1.33)	0.64	-	-
BMI (kg/m^2^)	1.04 (0.96, 1.12)	0.33	-	-	0.93 (0.81, 1.07)	0.31	-	-
Hypertension	0.98 (0.56, 1.72)	0.96	-	-	0.57 (0.23, 1.45)	0.24	-	-
Diabetes	1.40 (0.77, 2.55)	0.27	-	-	0.49 (0.14, 1.69)	0.26	-	-
Prior hysterectomy	1.48 (0.66, 3.28)	0.34	-	-	3.30 (1.18, 9.19)	0.02	2.58 (0.91, 7.28)	0.07
Prior POP surgery	0.80 (0.25, 2.57)	0.71	-	-	1.56 (0.36, 6.74)	0.56	-	-
Cystocele stage	1.09 (0.73, 1.61)	0.68	-	-	0.86 (0.45, 1.64)	0.64	-	-
Apical prolapse stage	0.93 (0.73, 1.19)	0.56	-	-	0.84 (0.57, 1.24)	0.38	-	-
Pad weight (g)	0.99 (0.98, 1.00)	0.12	-	-	1.00 (0.99, 1.01)	0.45	-	-
Qmax (mL/s)	1.02 (1.00, 1.05)	0.07	1.03 (1.00, 1.07)	0.04	1.01 (0.97, 1.06)	0.59	-	-
Voided volume (mL)	1.00 (1.00, 1.00)	0.48	-	-	1.00 (1.00, 1.00)	0.46	-	-
Post-void residual (mL)	1.00 (1.00, 1.00)	0.74	-	-	1.00 (0.99, 1.00)	0.54	-	-
PdetQmax (cmH_2_O)	0.97 (0.95, 1.00)	0.02	0.98 (0.96, 1.01)	0.15	0.99 (0.96, 1.02)	0.53	-	-
Detrusor overactivity	0.43 (0.15, 1.21)	0.11	-	-	1.83 (0.64, 5.28)	0.26	-	-
MUCP (cmH_2_O)	0.99 (0.98, 1.00)	0.01	0.99 (0.98, 1.01)	0.38	1.00 (0.99, 1.01)	0.90	-	-
SUI	1.12 (0.64, 2.01)	0.68	-	-	1.63 (0.66, 4.01)	0.29	-	-
OAB	1.06 (0.55, 2.04)	0.86	-	-	3.13 (1.27, 7.71)	0.01	3.22 (1.30, 7.96)	0.01
VD	1.38 (0.77, 2.49)	0.28	-	-	1.17 (0.44, 3.07)	0.75	-	-
VTH	0.59 (0.28, 1.25)	0.17	-	-	0.16 (0.02, 1.22)	0.08	0.19 (0.02, 1.46)	0.11
Mid-urethral sling	0.13 (0.02, 0.92)	0.04	0.15 (0.02, 1.17)	0.07	1.85 (0.61, 5.58)	0.27	-	-
Surgeon §								
A (reference)	1.00	-	1.00	-	1.00		-	-
B	0.98 (0.54, 1.78)	0.94	0.99 (0.44, 2.02)	0.98	0.70 (0.25, 1.99)	0.51	-	-
C	0.31 (0.09, 1.02)	0.054	0.23 (0.03, 1.79)	0.16	0.56 (0.12, 2.50)	0.45	-	-
D	6.28 × 10^−17^ (0, infinity)	1.00	-	-	1.69 × 10^−16^ (0, infinity)	1.00	-	-
E	6.27 × 10^−17^ (0, infinity)	1.00	8.30 × 10^−19^ (0, infinity)	1.00	1.70 × 10^−16^ (0, infinity)	1.00	-	-

CI = confidence interval. HR = hazard ratio. The other abbreviations are the same as in Table 1. † Cox proportional hazards model. ‡ Multivariable backward stepwise Cox proportional hazards modeling was performed using all variables with *p* < 0.10 in the univariate analysis. § Experienced POP surgical cases: surgeon A > surgeon B > surgeon C > surgeon D > surgeon E.

**Table 4:** Factors predicting postoperative voiding dysfunction (*n* = 206).

	Univariate	
Variables ‡	Odds Ratio (95% CI)	*p* †
Transobturator fixation	0.28 (0.03, 2.32)	0.24
Age (years)	1.02 (0.95, 1.11)	0.57
Parity	1.05 (0.57, 1.93)	0.89
BMI (kg/m^2^)	0.90 (0.72, 1.13)	0.35
Hypertension	0.96 (0.23, 3.95)	0.96
Diabetes	1.60 (0.37, 6.92)	0.53
Prior hysterectomy	1.09 (0.13, 9.24)	0.94
Prior POP surgery	1.74 (0.20, 15.12)	0.61
Cystocele stage	0.76 (0.26, 2.26)	0.62
Apical prolapse stage	1.50 (0.72, 3.13)	0.27
Pad weight (g)	1.01 (1.00, 1.02)	0.26
Qmax (mL/s)	0.97 (0.90, 1.05)	0.42
Voided volume (mL)	1.00 (0.99, 1.00)	0.18
Post-void residual (mL)	1.00 (0.99, 1.01)	0.46
PdetQmax (cmH_2_O)	1.02 (0.98, 1.06)	0.39
Detrusor overactivity	1.61 (0.30, 8.72)	0.58
MUCP (cmH_2_O)	1.00 (0.98, 1.02)	0.94
SUI	1.79 (0.43, 7.37)	0.42
OAB	2.04 (0.47, 8.87)	0.34
VD	2.54 (0.61, 10.49)	0.20
VTH	0.46 (0.06, 3.83)	0.47
Mid-urethral sling	2.31 (0.44, 12.06)	0.32
Surgeon §		
A (reference)	1.00	-
B	0.70 (0.13, 3.73)	0.68
C	0.69 (0.08, 6.14)	0.74
D	-	-
E	-	-

CI = confidence interval. The other abbreviations are the same as in Table 1. † Logistic regression analysis. ‡ Menopause = 1 predicts postoperative voiding dysfunction perfectly, the variable “menopause” was omitted. § Experienced POP surgical cases: surgeon A > surgeon B > surgeon C > surgeon D > surgeon E.

**Table 5:** Baseline and postoperative low urinary tract symptoms (*n* = 206).

	Transobturator (*n* = 68)			Sacrospinous (*n* = 138)		
Variables	Baseline	After Surgery	*p* †	Baseline	After Surgery	*p* †
SUI ‡	20 (33)	13 (21)	0.11	36 (31)	35 (30)	0.89
OAB	13 (19)	8 (12)	0.20	35 (25)	12 (9)	0.0001
VD	15 (22)	1 (1)	0.0005	45 (33)	7 (5)	<0.0001

Values are expressed as numbers (percentage). The abbreviations are the same as in Table 1. † McNemar’s test. ‡ Women who underwent concomitant mid-urethral slings are excluded from statistical analysis for SUI. Thus, there was 61 women in the transobturator fixation group and 118 women in the sacrospinous fixation group.

## Data Availability

The datasets of the current study are available from the corresponding author on reasonable request.
